# Lecture on Acute Rheumatism—Method of Examining Patients

**Published:** 1840-03-07

**Authors:** W. W. Gerhard


### CLINICAL LECTURE.

### PHILADELPHIA HOSPITAL.

*Wednesday, February 26th*, 1840.

### LECTURE ON ACUTE RHEUMATISM—METHOD OF EXAMINING PATIENTS.

By W. W. Gerhard, M. D.

*No.* 15—*Winter Course.*

I shall, to-day, point out a few cases of disease, and afterwards briefly recapitulate some of the points which have been touched upon in the course of clinical lectures, which this day brings to a close.

Last week you saw two cases of acute rheumatism, which I present again, in order that you may observe the progress of the affection, and the effects of some of the remedies which have been employed. Both of the patients have done well. The woman is almost recovered of the rheumatism proper; the arms and hands are scarcely at all swollen, and the motions of all the affected joints are easy and free from pain. The actæa racemosa has been fully tested in this case; and though its constitutional action was apparent, from the cerebral uneasiness which it produced, a very slight effect upon the disease was at any time been perceptible. After the failure of this article, cups were applied with the happiest effects. After convalescence had commenced, a slight increase of pain and swelling took place, which was readily arrested by cupping. This woman may now be considered well; but how long she will so continue, is another question. Acute rheumatism is extremely apt to relapse after convalescence has begun, and to return after a complete cure. Our patient is now probably past the danger of a relapse, but she must expect to suffer repeatedly from renewed attacks of the disease. It is found by experience, that these returns of rheumatism usually occur pretty regularly at certain periods of the year; that is, in the winter and spring months. When the disease is thus established by frequent recurrence at certain periods, the only measure which is likely to result in any permanent benefit, is a change of climate, which places the patient out of the influence of those circumstances on which the attacks of the disease depend.

I have said that the woman is cured of rheumatism; but she is not yet rid of its effects. There still remain a roughened sound, and disordered impulse of the heart, which indicate the persistence of endocarditis. This is declining very gradually, and will probably be distinguishable for some time to come; but I have no doubt that it will finally disappear without any permanent bad consequences. In relation to the duration of this heart disease depending on acute rheumatism, we have three varieties:—1. The inflammation of the heart may disappear with or before the rheumatism. 2. It may continue unabated for a great length of time, and result in incurable organic disease. 3. It may remain after the affection of the joints has disappeared, and then gradually decline, without producing any serious lesion of structure. Of this course of things I saw an illustration in the case of a woman, who was confined in our wards with acute rheumatism some years since. She presented the usual signs of endocarditis, which persisted for a year after the original affection disappeared; they then subsided of themselves, and the heart disease appeared not to have the slightest effect upon the subsequent state of the woman's health. But there can be no doubt that adhesion had taken place between the two surfaces of the pericardium, and that the endocardium had become more or less thickened.

The other rheumatic patient has also been treated with the cimicifuga, with partial benefit. It has appeared to favour the natural diaphoretic tendency of the complaint, and to produce something like a sedative effect. But in neither this nor the preceding case, has it exhibited those specific virtues which have been attributed to it, and in neither case has it been very efficient in bringing about the favourable result. In the woman especially, where we had the most indubitable evidence of the action of the remedy, it had very little effect in mitigating the disease. Hence other remedies had to be substituted for it. In the case now before us, Dover's powder was tried, together with the repeated application of leeches to the knee, and cups to the spine. These measures proved of great benefit as palliatives, but have not been adequate to the arrest of the disease. The rule which I laid down at the last lecture, holds good almost universally, viz., that no remedy can be relied on to arrest the course of acute rheumatism. Various means may be employed as palliatives, but they are palliatives only. In some cases, however, where there are shooting pains from the spine to the affected joint, cups applied to the spine may arrest the disease at once, or very soon; but this result is by no means to be anticipated in the great majority of instances. This patient has not thus far exhibited any signs of cardiac disease. You will hence conclude, that this is not of uniform occurrence in rheumatism; but, in those cases where there is high fever, it is rarely or never absent.

There are several other cases of acute rheumatism in the wards, which my time will not allow me to show you. I may remark, howdver, that they are of a mild kind, and have been treated by cupping, with the addition, in some cases, of sulphur and guaiacum, (a most valuable combination,) and in others, of the actæa racemosa, the powers of which I am desirous of putting to the fullest test.

I next present a case of phthisis following intermittent fever, which possesses some points of interest. The patient laboured under intermittent fever through the whole of last summer, but the cough commenced very lately. On his entrance into the hospital, I found that his physiognomy presented a mixture of the characters belonging to both diseases. An eye practised in such observations, will detect in this man's countenance the dusky aspect and harsh skin of phthisis, together with a slight yellowness, which is evidently the result of the intermittent,—the liver being more or less affected in nearly all cases of this fever. In miasmatic countries, it is a very frequent circumstance to observe phthisis following intermittent and remittent fevers. These diseases lead to its occurrence by debilitating the system, and placing it in the most favourable condition for the development of a tuberculous diathesis. Cases of pulmonary consumption occurring under such circumstances, are often classed under the head of liver diseases. It is no doubt true, that many cases of chronic organic disease of the liver are consequent upon autumnal fevers, but it is equally true that these fevers often give rise to phthisis, in constitutions predisposed to it. When phthisis is brought about in this manner, it should receive the earliest attention from the physician. If taken in its incipient stage, we may sometimes succeed in arresting it, by removing the condition of the system which has led to its development. By correcting the functions of the liver and alimentary canal, and restoring the blood to a healthy state, we get rid of the morbid condition which lies at the foundation, and it is possible, that the formation of tubercles may be suspended, unless cavities have already been formed. I say it is possible; for the most judicious treatment often fails. But this is principally in those cases which depend rather on a general vice of the economy than on any particular lesion. These disorders appear gradually, and without an appreciable cause. In the same manner, cancer, when dependent on a hereditary diathesis, is incurable by medicine or the knife. Diseases of the heart occurring in gouty people, afford another illustration. In such persons, particularly if advanced in life, there is a general disposition to the formation of calcareous concretions in various parts of the body; if they take place in the valves or lining membrane of the heart, a disorder of this organ is the result, which it is necessarily impossible to cure. But where any of the diseases mentioned arise from a strictly local lesion, it is possible to arrest them, provided the morbid action has not progressed beyond a certain stage.

I may further illustrate these remarks by reference to another patient. This man has hemiplegia, which was originally consequent upon apoplexy, and, of course, occurred suddenly. But it was not complete until a few days since, when he had another attack of apoplexy. The paralysis of the right side is now perfect, as is proved by the insensibility and rigidity of the right arm and leg, and the distortion of the mouth towards the left side. You must at once perceive, that this case is now incurable; an organic lesion of the brain has taken place, which the efforts of art would be insufficient to repair. But suppose that this case had been attended to before the incurable lesion took place, and when there was simply congestion of the brain without effusion, there is no doubt that it might have been arrested. The apoplectic hæmorrhage is merely an effort of nature, to relieve the congested vessels of the inordinate quantity of blood, and if we can remove such congestion by proper remedies, we fulfil the designs of nature, and at the same time avoid the injurious consequences which would follow her efforts, if unrestrained. In the same manner, where paralysis depends on inflammation of the membranes or of the substance of the brain, it is curable, so long as there is no lesion of structure. Thus the list of incurable diseases may be reduced to a very brief one: many of those usually considered incurable, are in fact not so, if they be attended to at the proper time. It is only those which depend on some constitutional vice, that must be reckoned incurable at all periods of their course.

I might exhibit several other cases of interest, but as this is the last lecture which I shall deliver, I think it more advisable to recapitulate some of the points which have been attended to during the course, more particularly in relation to diagnosis, and the degree of certainty which it has attained.

These lectures have been confined almost exclusively to the consideration of organic diseases. I have laid little stress on functional disorders, especially that extensive class coming under the head of neuralgiæ. My object has been to cultivate your habits of observation and of medical reasoning, to show you signs in their connexion with lesions. After you have attained a certain degree of knowledge on this subject, it will be very easy to investigate and understand simple functional disorders.

An important question occurs at the very commencement of the study of diagnosis, viz.: how should the physician conduct the examination of a patient, with the view of arriving most easily at the true nature of his disease. You have seen very little of methodical examination, in the very partial inquiries which have been made in your presence, (for my object in these lectures has been to teach, not to inquire,) and it may, therefore, be useful, to give you a few directions for conducting such examinations. It is always best to follow a certain order of investigation, and our attention should first be directed to the general appearance of the patient. We are often able at a glance to fix the disease with a considerable degree of certainty. The mode of walking, is one of those things which are observed at first sight, and it may of itself interpret the case.

Thus if the man limps, we suspect the existence of hemiplegia; if he walks with an unsteady gait, and drags both legs along with difficulty, we are pretty sure that the patient has paraplegia. If the patient is in bed, the position in which he is lying should also be carefully noted. The degree of strength he may possess is also of great importance, as it aids materially in distinguishing between acute and chronic diseases: in the former, it is more or less completely lost, whereas in the latter, it may remain almost unimpaired for a great length of time.

The next point to be attended to, is the general aspect and expression of the countenance. A practised eye will almost always discover something in the face, which is characteristic of the existing disease, that is, if it be of any severity. Thus in the last patient, the remarkable distortion of the mouth at once established the existence of paralysis; and in the preceding one, the countenance presented the characters of two diseases combined. The face likewise expresses the degree of dyspnœa, of pain, &c.: and the state of its capillary circulation often indicates with great certainty, the condition of the lungs, as well as of other organs, and the system generally. Thus in pneumonia, we have a circumscribed flush of a purple colour. In phthisis, we have also a circumscribed flush, but it is of a bright red. In apoplexy, the face is turgid with blood. In fevers there is a general flush, which is particularly marked in typhus fever. The eyes also present many appearances which are all important in diagnosis: but it is impossible for me even to glance at the various signs which may be detected in different portions of the countenance.

Having thus remarked the general appearance of the patient, which will in most cases give you a clue to the nature of the disease, you should inquire into the history of the complaint,—its mode of origin, its progress and duration. The certainty with which these points can be made out, will depend in a great measure upon the intelligence of the patient: sometimes he is naturally stupid, sometimes in a state of actual insensibility, and in such cases we have to depend upon the imperfect information derived from others. We should inquire into the history of each particular symptom, and the condition of the various functions at different periods of the disease. It is best to begin with the functions of the brain, and go regularly down, inquiring into the disorders of respiration, digestion, defecation, their duration, &c.

We are thus brought to the actual condition of the patient, which must be carefully inspected. The state of the mental faculties, of the senses, &c., must be inquired into. Next, the condition of the different parts of the alimentary canal is to be noted: the character of the tongue, the appetite, thirst and digestion, the mode of defecation, and the character of the fœces, the appearance of the urine, and the condition of every abdominal organ in succession. The thorax, from motives of convenience, is generally the last part examined. We inquire first into the functional disorders of its various organs. The action of the heart and the pulse demand great attention. The particular characters of the pulse, as to frequency, fulness, hardness, &c., are to be noted. But I lay much less stress on the pulse than many of my brethren do. It is worthy of attention as a collateral means of diagnosis, but is not so important as some persons would have us believe, particularly since we are possessed of more certain means of diagnosis. The pulse is more important as a guide in relation to certain therapeutic applications, than it is in the diagnosis of the disease. Attention is next to be paid to the cough, if there be any; it is found to possess very different characters in the various diseases of the chest. The expectoration is to be carefully examined, as to its frequency, the manner in which it is effected, and the appearance of the matters expectorated,—whether they be mucous, purulent, nummular, &c. The frequency of the respiration, and the manner in which it is performed, are important signs, particularly in diseases of the chest, and often aid materially in our prognosis. Thus, in pneumonia, if the respiration be very frequent and laboured, we know that the inflammation is one of grave character; but if slowly and naturally performed, our prognosis is favourable. Finally, we examine the lungs, one after the other, and the heart, by means of percussion and auscultation, which reveal to us the physical conditions of these organs.

Having thus investigated the history of the complaint, and the actual condition of the patient, by a comparison of these two sources of information, we make out our diagnosis. It will not do to depend upon the present state of things alone; nothing can be more uncertain in many cases, unless to a physician of very great experience in such investigations. An exclusive reliance upon it, is one of the most frequent errors among young practitioners. I repeat it, that a comparison of the present state with the previous history of the case, is essential to a correct diagnosis. There are in addition, various collateral circumstances which are of great importance. These relate to the age and sex of the patient, residence, habits of life, family, &c.

There can be no doubt that diagnosis has of late years attained a far greater degree of perfection than it before possessed; and its improvement has related more particularly to the connexion of signs with local lesions. But the art of prognosis has not been advanced in the same proportion, because it requires for its perfection, a more extended experience, continued through a long series of years. The diagnosis of local lesions is more certain, and will continue to improve every day, because the moderns pursue the most positive means of arriving at this end, viz., the comparison of signs with each other, and with the lesions discovered after death. This comparison of signs and lesions is essential to the elucidation of all diseases of an obscure character; and those of you who will pursue such a method of investigation, may render an important service to science, by unfolding the nature of certain diseases, which are as yet but imperfectly understood. For example, congestive fever is still involved in much obscurity. It is now known to be a variety of malignant intermittent or remittent. But it assumes a great variety of forms, and its distinctive characters have yet to be determined by a comparison of the observations of different persons, or of the same person through many successive years. The same thing was true, until recently of typhus or typhoid fevers. These distinct affections were formerly described under the same name, and insuperable difficulties were experienced by the student, in reconciling the discrepant accounts which were the necessary consequence of this confusion. But the diseases are now known to be altogether distinct, and the diagnosis between them has been rendered quite easy.

The points of diagnosis and pathological anatomy which have been insisted on during the course, cannot but aid you considerably in the study of disease, and give you more precise ideas of their essential nature. The demonstrations of morbid specimens which have been made from time to time, will also enable you to individualize the descriptions of organic lesions which you will find in different works. But a perfect acquaintance with any of these subjects cannot be attained, except by a long course of practice, and a careful observation.

The application of the principles of therapeutics, is another most important subject of study. In fact, this is the great object of the practical physician, and diagnosis and pathology are for the most part preparatory to it. In order to understand this important subject, it is necessary to be well versed in diagnosis, and to have at your command those remedies which are best adapted to the relief of ordinary, as well as of extraordinary cases of disease. You must also, possess the means of combating any particular symptom or complication, however slight or temporary these may be. This branch of medical science has been much improved of late, but it is yet far from being so certain as diagnosis; for the action of remedies is constantly influenced by the season, the age and constitution of the patient, and a thousand other circumstances, which it is often difficult to appreciate. There are many diseases which you will have little difficulty in curing; in fact, you can hardly prevent them from terminating favourably. These diseases are such as have a natural termination, and in such, it ought to be your object, not so much to interfere with the disease, as to prevent the occurrence of certain subsequent lesions to which it may give rise, if it is turned aside from its natural course. It is therefore necessary to watch the progress of such cases, and see that nothing interferes with their favourable tendency. It is wrong to harass the patient with unnecessary remedies, and thus, as it were, turn nature out of doors. It is as important that you should know when not to act, as when to act. Typhoid fever is one of those diseases which have a natural termination; but intermittent and remittent fevers have not. More active treatment is therefore demanded in the latter than in the former. In like manner, as I have already told you, there are some diseases of the chest which have a natural course and termination, while others have not. If time permitted, I might adduce many similar instances from other classes of diseases. But this is a subject upon which I have already enlarged sufficiently during the progress of the course.

As you are aware the present lecture brings this course to a close, the time allotted to it is so short, and so much interrupted by the numerous engagements which press upon you during the winter months, that it has been limited to but a few of the many subjects properly pertaining to a course of clinical medicine. In selecting these subjects, I have necessarily laid the greatest stress upon the diseases which the season rendered most abundant. And although many have been omitted, partly from the want of suitable illustration, but much more from the difficulty of saying more in so short a time, we have demonstrated to you the characteristic symptoms of the most frequent diseases, and have illustrated the methods of investigation. What is true in this respect of one, is applicable to all; nature is the same, she puts on different garments, but with attention, strengthened by a knowledge of the subject, you may divest the truth of all unnecessary concealments. The oft repeated maxim that truth is in things themselves, and not in our opinions, is applicable to clinical medicine, more than to most other subjects of inquiry.

I trust that you have become familiar both with the means of investigation and the habits of careful, thoughtful comparison, which may serve you in the future pursuits of your profession. These are the only true foundations for correct medical observation, or successful medical practice.

Note.—These lectures were delivered once in every week during the winter, including a period of seventeen weeks. The first lecture was general in its character, and was not reported. One was omitted from the day appointed falling upon Christmas. Fifteen remain, and constitute the published course.

